# PD54 Impact Of Experiencing Vaso-Occlusive Crisis In Patients With Sickle Cell Disease: Systematic Review And Meta-Analysis Of Prognostic Studies

**DOI:** 10.1017/S026646232400312X

**Published:** 2025-01-07

**Authors:** Bruna Ascef, Mariana Oliveira Marques, Diego Kashiura, Juliano Bertinato, Haliton Alves De Oliveira

## Abstract

**Introduction:**

Sickle cell disease (SCD) is characterized by recurrent painful ischemic vaso-occlusive episodes (VOEs). Acute episodes of pain, often termed sickle cell pain crises or vaso-occlusive crises (VOCs), are one of the most common and debilitating manifestations of SCD. Here we present a systematic review of 31 trials of 202,758 patients with SCD.

**Methods:**

This study followed the guidelines of Riley et al. for conducting a systematic review and meta-analysis of prognostic factor studies and was reported according to PRISMA guidelines. Literature searches were conducted in the PubMed, Embase, and LILACS databases. The websites of the Annual Academy for Sickle Cell and Thalassemia Conferences, the European Hematology Association, and the American Society of Hematology were also searched. Additionally, manual searches were conducted in Google Scholar, Epistemonikos, and the reference lists of included studies. The searches were performed on 22 March 2023.

**Results:**

In total, 31 studies were included in this systematic review. There was considerable heterogeneity in the definition of prognostic factors of interest across the included studies. Findings revealed a link between VOCs and reduced health-related quality of life (HRQoL), severe pain, and a high rate of hospitalization. Although VOCs were related to an increased mortality risk, the mortality rate remained relatively low, with acute chest syndrome being a common cause of death. Despite the study heterogeneity, consistent evidence highlighted the impact of VOCs on SCD-related hospitalizations (pooled hospitalization rate due to VOEs of 47%, 95% confidence Interval: 33, 61; 16 studies, 139,752 participants).

**Conclusions:**

This study suggests that VOCs reduce HRQoL, cause severe pain, and lead to high rates of hospitalization in patients with SCD. Furthermore, VOEs were related to an increased mortality risk. Future research should prioritize more well-designed comparative prospective studies and standardization of the definition of VOCs. A better understanding of VOCs as a prognostic factor could enhance HRQoL, alleviate healthcare system burden, and inform more effective interventions for patients with SCD.

